# Characterization of *Lavandula* spp. Honey Using Multivariate Techniques

**DOI:** 10.1371/journal.pone.0162206

**Published:** 2016-09-02

**Authors:** Leticia M. Estevinho, Emerson Dechechi Chambó, Ana Paula Rodrigues Pereira, Carlos Alfredo Lopes de Carvalho, Vagner de Alencar Arnaut de Toledo

**Affiliations:** 1 Departamento de Biologia e Biotecnologia, Escola Superior Agrária, Instituto Politécnico de Bragança, Bragança, Portugal; 2 Centro de Ciências Agrárias, Ambientais e Biológicas, Universidade Federal do Recôncavo da Bahia, Cruz das Almas, Bahia, Brazil; 3 Departamento de Zootecnia, Universidade Estadual de Maringá, Maringá, Paraná, Brazil; Islamic Azad University Mashhad Branch, ISLAMIC REPUBLIC OF IRAN

## Abstract

Traditionally, melissopalynological and physicochemical analyses have been the most used to determine the botanical origin of honey. However, when performed individually, these analyses may provide less unambiguous results, making it difficult to discriminate between mono and multifloral honeys. In this context, with the aim of better characterizing this beehive product, a selection of 112 *Lavandula* spp. monofloral honey samples from several regions were evaluated by association of multivariate statistical techniques with physicochemical, melissopalynological and phenolic compounds analysis. All honey samples fulfilled the quality standards recommended by international legislation, except regarding sucrose content and diastase activity. The content of sucrose and the percentage of *Lavandula* spp. pollen have a strong positive association. In fact, it was found that higher amounts of sucrose in honey are related with highest percentage of pollen of *Lavandula* spp.. The samples were very similar for most of the physicochemical parameters, except for proline, flavonoids and phenols (bioactive factors). Concerning the pollen spectrum, the variation of *Lavandula* spp. pollen percentage in honey had little contribution to the formation of samples groups. The formation of two groups regarding the physicochemical parameters suggests that the presence of other pollen types in small percentages influences the factor termed as “bioactive”, which has been linked to diverse beneficial health effects.

## Introduction

Lavender is the popular name for the plants of the genus *Lavandula*, Lamiaceae family. This genus contains many species, among which several are grown extensively in temperate climates for ornamental purposes, for use as aromatic herbs or for oil extraction. In beekeeping, the *Lavandula* honey is greatly appreciated by consumers due to its pleasant aroma and flavour. Recently, several research studies have studied the physicochemical and sensory properties of this honey, as well as those related to their bioactive compounds [1, 2, 3, 4, 5].

The botanical origin and the physicochemical and sensory properties of honey are critical parameters that influence both product quality and market value. In Europe, the richness and diversity of melliferous flora, both from wild species and cultivated plants, may give rise to a variety of monofloral honeys [6].

The melissopalinogical and physicochemical analysis of honey have been the most frequently used to ensure that a particular type of honey receives the designation of monofloral and even a geographic certification [7, 8, 9, 10]. Quantitative pollen analysis provides important data for the honey characterization, especially with regard to geographical origin, occurrence of nectar plants and harvest seasons. The melissopalynology can provide information about the processes of filtration and extraction of honey, as well as the occurrence of fermentations, adulteration and contamination [11].

The identification of the botanical origin of honey is a difficult task, being all the currently used methodologies associated to errors and unambiguous results [12]. In general, when the pollen spectrum contains more than 45% of pollen of the same species, the so-called dominant pollen, the honey is classified as monofloral [13]. However, such classification cannot be applied to all plant species, since many types of pollen are considered representative even when present in very low amounts, as in the cases of *Lavandula* spp. [3], *Citrus* spp., *Rosmarinus officinalis* [14] and *Arbutus unedo* [15] honeys. Also, several researchers consider that the melissopalynological and physicochemical analysis by themselves are insufficient to enable a non-subjective identification of honey floral origin [16, 17].

Recently, the traditional techniques for monofloral honey characterization have been complemented with other analytical methods, among which the studies of volatile organic compounds using solid-phase microextraction-gas chromatography-mass spectrometry [6, 18], of honey’s sugars profile by HPAEC-PAD [19] and of flavonoid glycosides using HPLC-MSn [20].

In addition, for a better characterization of monofloral honey some authors combine multivariate statistical procedures to the physicochemical or bioactive compounds analysis [21, 22, 23, 24, 25]. However, for melissopalynological analysis, many statistical techniques may be unfeasible because usually the data do not follow a multivariate normal distribution. In such cases, the Non-metric Multidimensional Scaling (NMDS) may be the solution, because it can produce ordinations of objects in a reduced number of dimensions and from any distance matrix, being therefore a flexible technique that can fit different types of data [26, 27]. NMDS can model nonlinear relationships among variables, it can handle nominal or ordinal data and does not require multivariate normality. As such, NMDS appears as a sound alternative to methods like factor analysis and smallest space analysis [28].

The aim of this study was to characterize the monofloral honey of *Lavandula* spp. from different regions of Portugal using multivariate statistical techniques for melissopalynological, physicochemical and bioactive compounds data analysis.

## Material and Methods

### Ethics Statement

No specific permits were required for the described field studies. The sampling sites are not protected in any way and the field studies did not involve endangered or protected species.

### Geographic origin of honey samples

The study area included apiaries from different regions of Portugal: Chaves (n = 10), Fafe (n = 10), Mirandela (n = 10), Mogadouro (n = 10), Lousã (n = 10), Amieira (n = 12), Castelo-Branco (n = 10), Santarém (n = 10), Montemor-o-Novo (n = 10), Odemira (n = 10) and Monchique (n = 10), totalizing 112 *Apis mellifera* honey samples that were harvested by beekeepers and delivered to the laboratory, where these were kept at 25°C in the dark until analysis.

### Samples’ characterization

The physicochemical properties of honey samples were performed according to the methods previously described in detail [29, 30]. The evaluated parameters were: moisture (%), ash (%), electrical conductivity (mS/cm), hydroxymethylfurfural content (HMF) (mg/kg), free acidity (meq/kg), diastase activity (Schade units/g), reducing sugars (%), apparent sucrose (%), pH and proline (mg/kg). The protein content (mg/kg) was determined according to the method described by Nogueira et al. [31]. The total phenolic content of honey samples was estimated following the Folin–Ciocalteau method [32]. For the total flavonoid determination, a method described by Kim et al. [33] and modified by Al et al. [34] for honey sample was used. For each honey sample we performed three replicates of each parameter (S1 Dataset).

All the samples were subjected to pollen analysis by acetolysis method [35]. The examination of the pollen slides was carried out with a Leitz Diaplan microscope (Leitz Messtechnik GmbH, Wetzlar, Germany) at 400× and 1000× in order to make a sound identification of the pollen types. A minimum of 1000 pollen grains were counted per sample. To recognize the pollen types, it was used the reference collection from the CIMO-Mountain Research Center (Agricultural College of Bragança, Polytechnic Institute of Bragança) and different pollen morphology guides. The following terms were used for pollen frequency classes: predominant pollen (P, more than 45% of pollen grains counted), secondary pollen (S, 16–45%), important minor pollen (IM, 3–15%) and minor pollen (M, 1–3%) [13] (S2 Dataset).

### Statistical analyses

Mean, medians, percentiles, and standard errors of the means (SEM) for physicochemical parameters, bioactive compounds (total phenol and total flavonoid) and pollen data were calculated.

The data of the physicochemical variables, phenolic compounds and *Lavandula* spp. pollen percentage in honey were analysed by multivariate factor analysis. The pollen of *Lavandula* spp. was included in this analysis because not only was it found in all samples, but its relative frequency in honey was higher than 15%. Persano Oddo and Piro [1] and Gomes et al. [3] considered this percentage sufficient to characterize the honey as monofloral for *Lavandula* spp.

After we obtained the correlation matrix X’X, we performed a diagnosis of multicollinearity based on the condition number. The adequacy of the data for the multiple factor analysis was performed by using Bartlett's test of sphericity and the Kaiser-Meyer-Olkin measure of sampling adequacy. Regarding the technique of factor extraction, we used the principal component technique. The factors were extracted until we obtained the baseline of 60% of cumulative variance [36]. After we extracted the factor loadings, the factors were established by rotation by the varimax method.

The data obtained in the physicochemical and melissopalynological analyses of the honey were examined with Non-metric Multidimensional Scaling (NMDS), employing Euclidean distance after chord transformation. After we built the dissimilarity matrix with the normalized data, we used the command “metaMDS” to generate random and interactive processes to find the best solution possible. The goodness of fit measured of the NMDS was evaluated according to "stress" and Shepard diagrams. Next, we added the information from a result of clustering for ordering the NMDS. To do that, we calculated the clustering UPGMA of the dissimilarity matrix. For the estimate of the fitting between the dissimilarity matrix and the dendogram generated, we calculated the cophenetic correlation coefficient (CCC). After we identified the clusters, we tested the results of the physicochemical analyses using the t test of comparison of averages (*p*<0.05). All statistical analyses were performed using “R” statistical and programming environment version 3.0.2 [37].

## Results and Discussion

To assess the quality of Portuguese honey samples and to determine their botanical origin, we carried out physicochemical analyses and determined the concentrations of the bioactive compounds. In the analyzed honey samples, all physicochemical parameters fulfilled the general honey quality standards established by the European legislation [38, 39], apart from diastase and sucrose content. The diastase activity ranged from 4.55 to 25.21 Schade units (mean value of 12.02 ± 0.36 SEM), while the percentage of apparent sucrose was between 0.87 and 14.23% (mean ± SEM of 4.99 ± 0.31%). For the former parameter, 11% of the samples were not in agreement with the legislated limits; for the later the percentage was higher– 24%.

In general, international legislation of honey determines that diastase activity should be not less than 8 Schade units and the sucrose content not more than 5% [38, 39]. However, it must be noticed that for some particular honey types like monofloral from *Lavandula* spp., current norms and regulations allow values of diastase activity between 3 and 8 Schade units (since HMF content is inferior to 15 mg/kg) and contents of sucrose until 15% [38, 39]. As such, it can be assumed that the assessed honey samples were in accordance with the legislation, because the HMF values were lower than 15 mg/kg and the relative frequency of *Lavandula* spp. pollen higher than 15%, which is sufficient to consider the botanical origin of the honey as monofloral of this plant [1, 3].

The total phenols, flavonoids and proline concentrations presented great variations between samples, which can be confirmed by the highest estimates in the standard errors of the means. However, in spite of these differences, the content of proline was always bellow 180 mg/kg. This is important since according to Bogdanov [30] concentrations above that threshold could indicate adulteration or suggest premature honeys’ harvest. Considering the other parameters, except for the maximum and minimum values, 90% of the samples had less variation (Table 1). In fact, the amount and type of bioactive compounds depends largely upon the floral source/variety of the honey, seasonal and environmental factors, as well as conditions of processing and storage [40, 41].

**Table 1:** Summary of physicochemical parameters and bioactive compounds in honey samples from Portugal beehives.

Parameters^a^	High	Low	Median	5%tile	95%tile	Mean (SD)^b^	SEM^c^
Acidity (meq/kg)	39.65	2.80	25.93	15.81	36.05	25.72(6.74)	0.64
Ash (%)	0.54	0.04	0.16	0.08	0.42	0.20(0.11)	0.01
Conductivity(mS/cm)	0.86	0.16	0.31	0.20	0.75	0.37(0.17)	0.02
Diastase (Shade units)	25.21	4.55	11.80	7.06	18.71	12.02(3.84)	0.36
Flavonoids (mg/kg)	158.00	60.00	116.3	68.94	150.53	110.67(27.08)	2.56
HMF (mg/kg)	17.34	0.25	3.36	0.51	14.77	5.23(4.89)	0.46
Moisture (%)	19.12	14.25	16.30	14.96	18.71	16.53(1.09)	0.10
Ph	13.29	2.23	3.55	2.68	5.21	3.76(1.17)	0.11
Total phenols (mg/kg)	234.7	88.00	147.3	91.54	227.52	152.32(44.53)	4.21
Proline (mg/kg)	308.30	183.40	258.10	230.28	303.32	263.68(26.83)	2.54
Protein (mg/kg)	0.54	0.21	0.34	0.23	0.52	0.36(0.10)	0.01
Sucrose (%)	14.23	0.87	4.01	2.05	13.31	4.99(3.23)	0.31
Reducing sugars (%)	79.83	60.41	70.55	64.79	76.98	70.54(4.07)	0.38

^a^ The values of each parameter were obtained from the analysis of 112 honey samples.

^b^ Mean and Standard deviation (SD).

^c^ Standard errors of the means (SEM).

Factor analysis was performed to describe the original set of physicochemical variables in a smaller number of factors and to interpret through the factor loadings or model parameters the correlations between these and the original variables. The Kaiser-Meyer-Olkin sampling adequacy test was 0.61 and the Bartlett sphericity test was significant (*p*<0.001), indicating that the correlation matrix X'X is not an identity matrix and that there are significant sample correlations between the physicochemical variables, which is suitable for multivariate factor analysis [36]. The multicollinearity diagnosis indicated low collinearity (condition number < 100), so it was decided to not exclude any database variable.

Factor analysis indicated that 72.00% of the total variation of the physicochemical parameters can be explained by the overall effect of the main five factors. The first five factors have eigenvalues that correspond to 23.00%, 16.00%, 13.00%, 13.00% and 8.00% of the total variance (Table 2). Similar results were already reported by Marchini et al. [21] and Abadio-Finco et al. [22].

**Table 2:** Initial Eigenvalues and cumulative variance.

Component	Importance of components			Standardized loadings		
	standard deviation	% of variance	Cumulative (%)	SS loadings	% of variance	cumulative (%)
1	2.03	29.41	29.41	3.20	23.00	23.00
2	1.41	14.19	43.60	2.18	16.00	38.00
3	1.26	11.42	55.03	1.82	13.00	51.00
4	1.12	8.98	64.01	1.75	13.00	64.00
5	1.06	8.04	72.06	1.13	8.00	72.00
6	0.98	6.87	78.93			
7	0.95	6.47	85.40			
8	0.86	5.28	90.68			
9	0.74	3.94	94.62			
10	0.61	2.64	97.26			
11	0.47	1.42	98.68			
12	0.37	1.00	99.68			
13	0.18	0.24	99.92			

The final factor loadings, obtained by varimax rotation method, associated to each factor are presented in Table 3.

**Table 3:** Final factor loadings after rotation by varimax method.

Variable	Factor^a^				
	1	2	3	4	5
Moisture	0.435	0.216	0.381	**0.477**	0.234
HMF	0.115				**0.858**
Reducing sugar	0.378	0.229	0.389	**0.603**	0.118
Sucrose			**0.809**	-0.317	
Conductivity		**0.963**			
Acidity	0.319	**0.394**	-0.115	0.274	**-0.402**
pH				**0.623**	-0.123
Ashes		**0.968**			
Diastase		0.196	-0.206	**0.697**	
Proline	**0.978**				
Protein	0.22		0.247	0.326	**-0.357**
Total phenols	**-0.946**			-0.171	
Flavonoids	**-0.916**	-0.111			
*Lavandula* pollen			**0.822**	0.123	

^a^ Extraction method of factor loadings: principal component analysis. The five factors are established by varimax rotation method.

Factor 1, named "bioactive", was very strong and positively associated with the proline variable and very strong and negatively associated with the variables total phenols and flavonoids. Factor 2, designated as “minerals”, was strong and positively associated with electrical conductivity, acidity and ashes variables. Factor 3, “botanical origin”, had a higher positive association with the variables apparent sucrose and pollen of *Lavandula* spp. Factor 4, denominated “quality” was positively associated with the variables humidity, reducing sugars, pH and diastase. Finally, the factor 5, “other variables” had a strong positive association with HMF variable, and a negative association with the variables acidity and protein (Table 3).

The factor analysis was not able to reduce the number of original physicochemical variables to produce ordinations of sampling sites in a two-dimensional graphic to classify honey samples. So, it was decided to use the NMDS to express the relation between variables, as well as between the sampling sites and the variables and, then, the cluster analysis.

**Fig 1:**
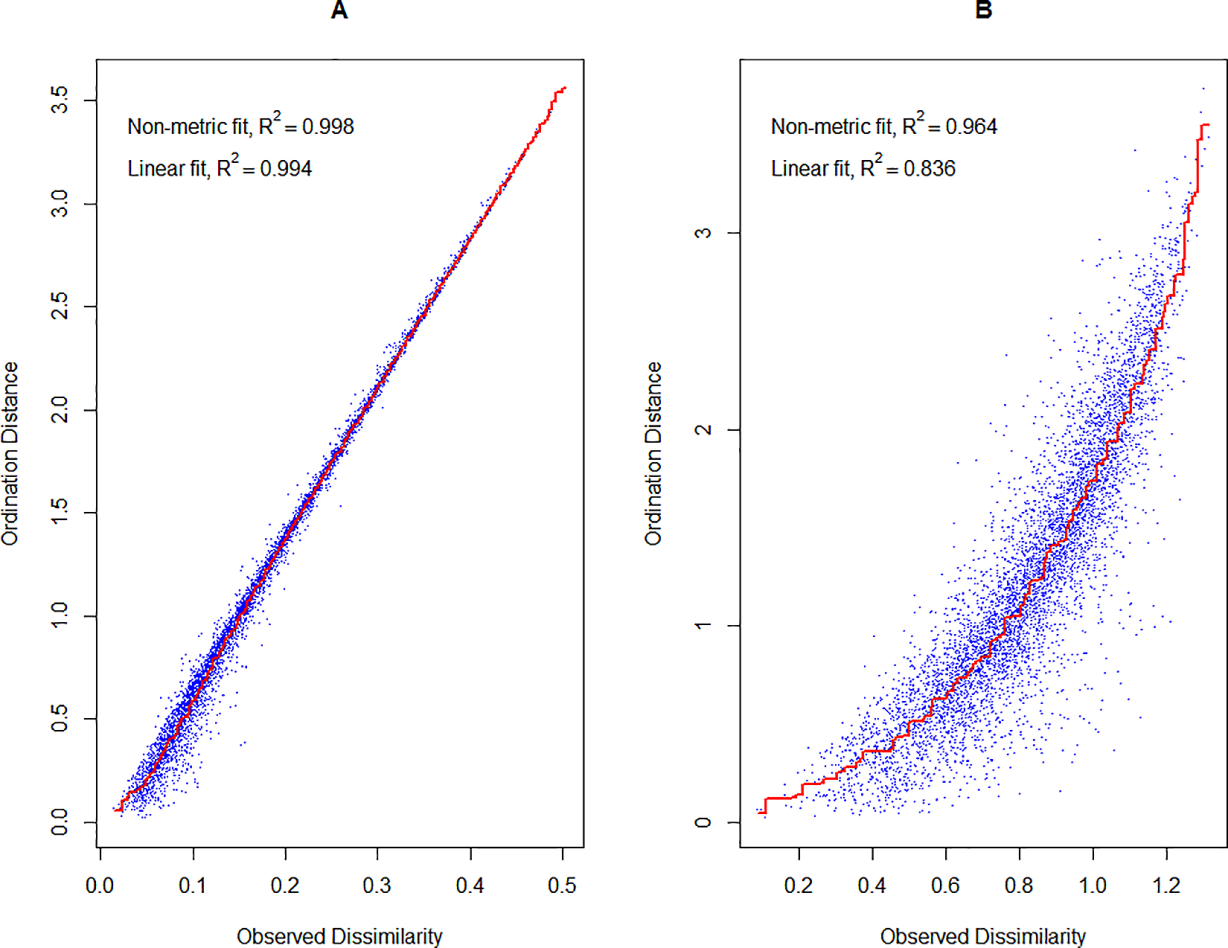
Shepard diagrams of the NMDS results. **(A) physicochemical data (normalized) and (B) melissopalynological data (normalized).** Dashed line signifies a perfect linear relationship between calculated and ordination distances.

The corresponding Shepard diagrams (Fig 1) indicate that as adjustment levels are raised, the distances portrayed in ordination space are more linearly related to those on which the calculations are based.

**Fig 2:**
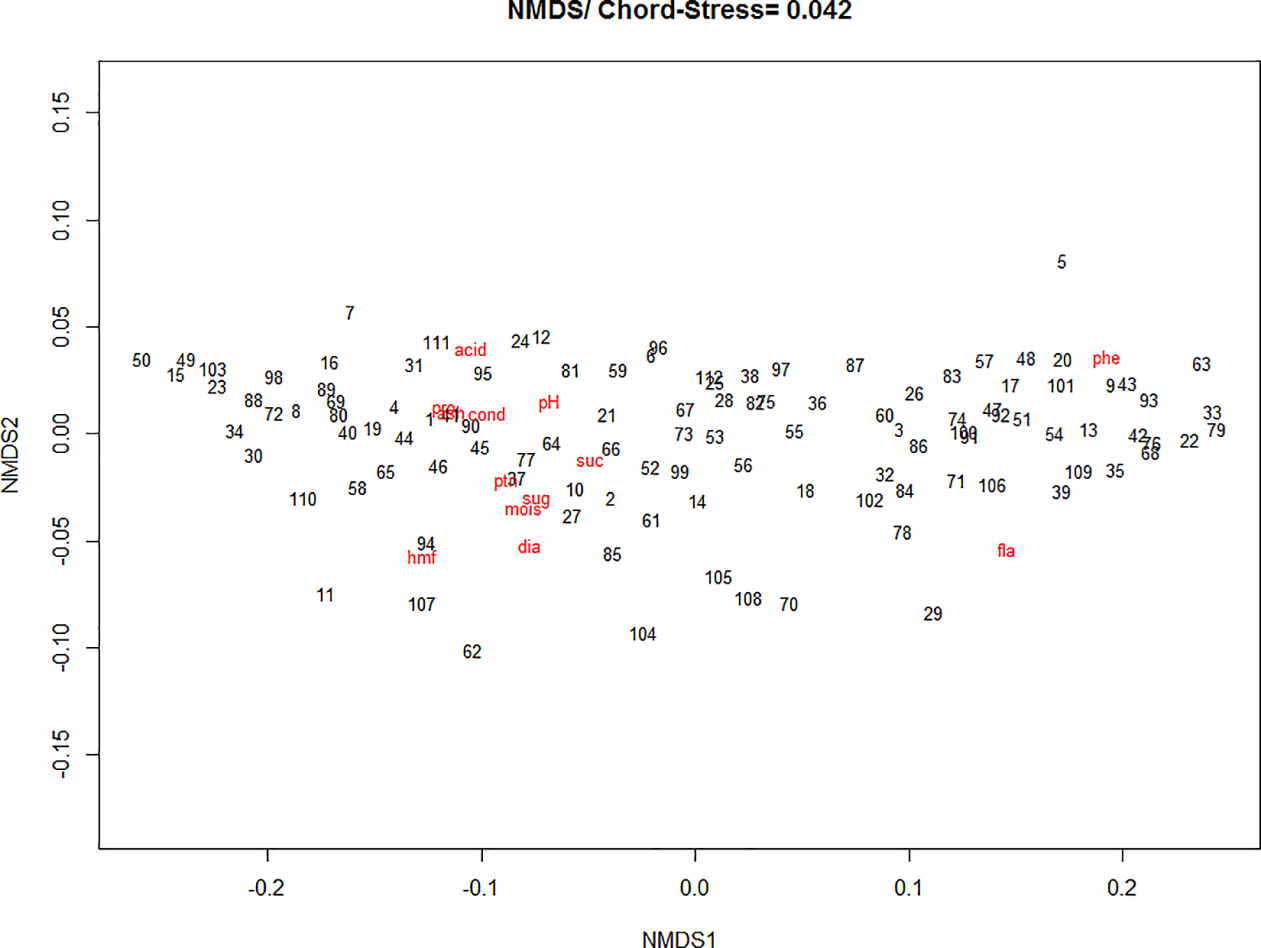
NMDS biplot of a chord distance matrix of the physicochemical data in honey (stress = 0.04). Sites: Mogadouro (1–10); Mirandela (11–20); Chaves (21–30); Castelo-Branco (31–40); Fafe (41–50); Lousã (51–60); Santarém (61–70); Odemira (71–80); Monchique (81–90); Montemor-o-Novo (91–100) and Amieira (101–112).

In Fig 2 is presented a more similar group of samples, located on the right and with higher values for phenols and flavonoids, which are more correlated to each other. A second group of similar samples located on the left in the ordination presented higher values for the other physicochemical parameters, which are more correlated between them. In Fig 2, the associations between the variables were similar to the factor analysis (Table 3), indicating the similarity between the techniques for the obtained answers.

**Fig 3:**
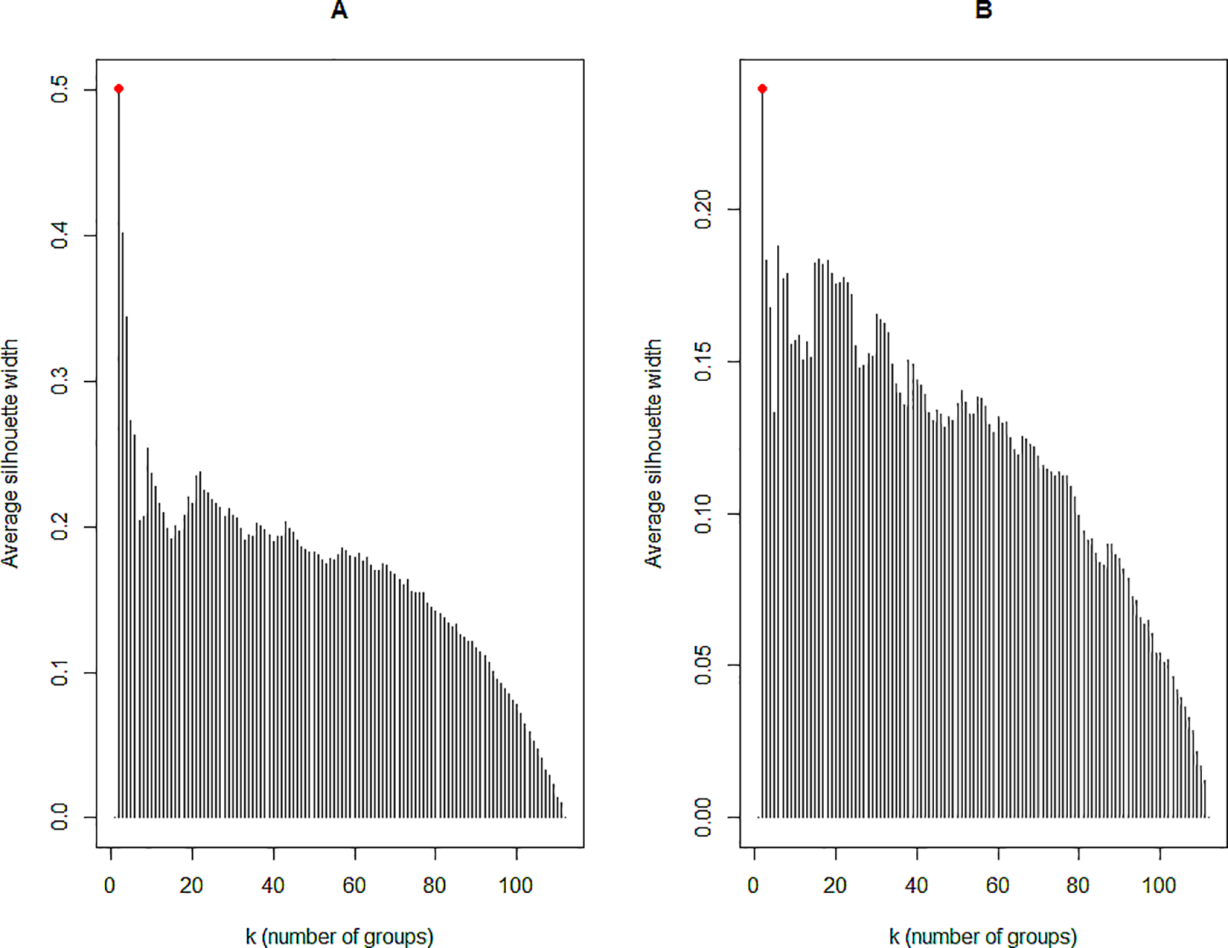
**Bar plots showing the average silhouette widths for physicochemical data (A) and melissopalynological data (B).** The best partition by this method is the one with the largest average silhouette width. For further details refer to [27].

**Fig 4:**
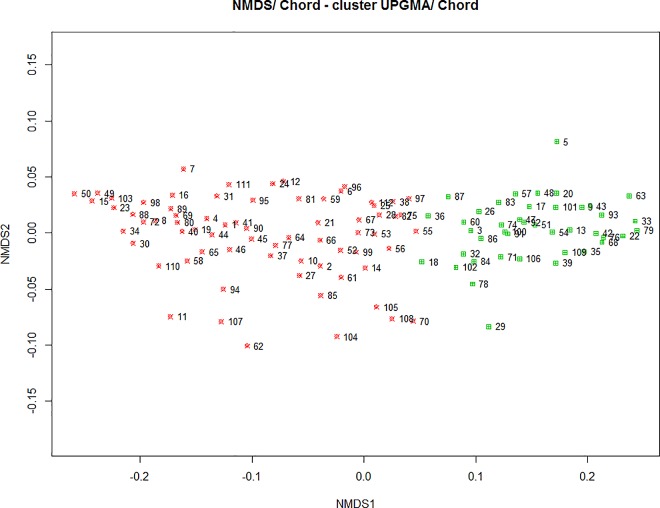
UPGMA clustering of a matrix of chord distance among sites in a NMDS ordination plot for physicochemical data (CCC = 0.72). Sites: Mogadouro (1–10); Mirandela (11–20); Chaves (21–30); Castelo-Branco (31–40); Fafe (41–50); Lousã (51–60); Santarém (61–70); Odemira (71–80); Monchique (81–90); Montemor-o-Novo (91–100) and Amieira (101–112).

We extracted two groups using the criterion of the silhouette width from the result of cluster analysis of honey samples in relation to physicochemical parameters (Figs 3A and 4). The cophenetic correlation coefficient was 0.72, which is reasonable as a factor of hierarchical representativity [42].

In Fig 4 it can be observed that the green group on the right differed significantly from the red located on the left regarding the contents of flavonoids (*p* = 0.002) phenols (*p* = 0.0001) and proline (*p* = 0.0001). On the other hand, it were not detected differences amongst groups for the other physicochemical parameters, namely concerning protein content (*p =* 0.42) and sacarose (*p =* 0.30) (Table 4). This highlights the great similarity amongst most of the samples, except for the "bioactive" factor extracted by factor analysis (Table 3). Also, it suggests that such differences on this factor may be due to the presence of other botanical families of pollen apart from *Lavandula* spp. (non significative, *p* = 0.273).

**Table 4:** Comparison between the groups extracted from the UPGMA cluster analysis of the chord distance matrix for physicochemical parameters of honey.

Variables	Grup 1 (n = 42)	Grup 2 (n = 70)	t	*p-value*
Lavandula pollen (%)	36.27 ± 15.55	33.17 ± 10.69	1.09	0.273
Acidity (meq/kg)	24.99 ± 5.63	26.08 ± 7.23	-0.85	0.422
Ash (%)	0.19 ± 0.12	0.21 ± 0.11	-0.77	0.44
Conductivity (mS/cm)	0.36 ± 0.19	0.38 ± 0.16	-0.46	0.64
Diastase (°Gothe)	12.35 ± 3.67	11.85 ± 3.94	0.64	0.52
Flavonoids (mg/kg)	134.59 ± 14.80	98.87 ± 23.80	0.39	0.002*
HMF (mg/kg)	4.75 ± 4.51	5.47 ± 5.07	-0.74	0.46
Moisture (%)	16.44 ± 1.14	16.58 ± 1.07	-0.64	0.52
pH	3.90 ± 1.73	3.69 ± 0.77	0.72	0.48
Total phenols (mg/kg)	194.14 ± 26.23	131.69 ± 36.51	9.28	0.0001*
Proline (mg/kg)	246.34 ± 21.51	272.23 ± 25.10	-5.37	0.0001*
Protein (mg/kg)	0.35 ± 0.09	0.36 ± 0.09	-0.81	0.42
Sucrose (%)	5.51 ± 3.97	4.76 ± 2.79	1.03	0.30
Reducing sugars (%)	70.68 ± 4.01	70.47 ± 4.13	0.26	0.79

The botanical origin is one of the factors that most influences the content of phenolic compounds [43]. Even though we observed significant differences amongst groups regarding the "bioactives" factor, this does not collide with the prior characterization of the samples as monofloral honey of *Lavandula* spp. Indeed, studies conducted by Gomes et al. [3] also described variations on the content of polyphenols and flavonoids between samples of monofloral honeys of *Lavandula* spp. harvested in Portugal, whilst the other physicochemical parameters were statistically not different. Estevinho et al. [10] also observed differences on the concentrations of phenols and flavonoids obtained for different samples harvested in different places from the same region and with identical botanical origin. In addition, Meda et al. [44] reported that the content of phenolics and proline differed significantly whether the samples were monofloral or multifloral.

Concerning proline, regardless of the group, our results corroborate the obtained using monofloral honeys from other underrepresented pollen types, among which *Rhododendron* (264 mg/kg), *Robinia* (222 mg/kg) and *Rosmarinus* (271 mg/kg). On the other hand, our data differed from the obtained with monofloral honeys from overrepresented botanical species, like *Castanea* (585 mg/kg), *Eucalyptus* (528 mg/kg) and *Helianthus* (562 mg/kg_)_ [1].

Proline, which may constitute up to 70% of the free amino-acid pool in pollen grains, has long been regarded as playing a pivotal role to pollen vitality and fertility. Also, it has also been reported to be associated with pollination, since appear to have a strong preference for proline-enriched nectars [45]. In this context, the differences observed between groups regarding proline content (*p* = 0.002) also support the presence of other polinic types apart from *Lavandula* spp. that, as described above, may influence the concentration of bioactive compounds.

The concentration of bioactive compounds determined in this study for the samples of monofloral *Lavandula* spp. honey was high. Polyphenols are naturally occurring compounds found largely on plants that are generally involved in defense against ultraviolet radiation, oxidative stress or aggression by pathogens, presenting pleiotropic health beneficial effects and potential therapeutic applications [46].

The amino acid composition of food protein hydrolysates has been reported as a major determinant of their antioxidant properties. Indeed, hydrophobic amino acids, among which proline, leucine, valine and methionine, have been considered as very important in the protection against oxidative stress [47] reason by which their determination is an important complement whenever antioxidant activities of a food product are under assessment [44]. In addition, the assessment of the interaction between peptides and other chemical components also allows a more complete approach of the biological systems and their subsequent application on the diagnosis and treatment of human diseases where free radicals are implicated [48].

In order to determine the nectar source of the honey samples, we performed melissopalyinological analyses. We identified 25 pollen types out of 112 samples that we analysed, being *Cistus* spp., *Echium* spp. and *Lavandula* spp. identified in more than 50% of the samples. Eleven pollen types, *Acacia* spp., *Cardus* spp., *Chamaespartium* spp., *Eucalyptus* spp., *Genista* spp., *Levatera* spp., *Medicago* spp., *Pinus* spp., *Quercus* spp., *Thimus* spp. and *Vicia* spp. were detected in less than 10% of honey samples. The pollen of *Lavandula* spp. was detected in all samples, being its percentage higher than 45% in 23 samples and between 16–45% in the remaining 89 honey samples. The percentage of pollen grains that were not *Lavandula* spp. was greater than 45% in only one sample. In this honey, the percentage of pollen from *Erica* sp. was 52.56% (Table 5).

**Table 5:** Summary of pollen analysis in honey samples from Portugal beehives.

Genus^a^	Number^b^ Samples detected	Detection (%)					
		High	Low	5%tile	95%tile	Mean (SD)^c^	SEM^d^
**Aca**	6 T, 4 S, 2 IM	0.00	30.93	0.00	3.53	0.92 (4.24)	0.40
**Ant**	11 T, 9 S, 2 IM	0.00	27.93	0.00	4.97	0.82 (3.21)	0.30
**Api**	18 T, 1 S, 15 IM, 2 M	0.00	18.72	0.00	6.29	1.04 (2.81)	0.27
**Car**	6 T, 5 IM, 1M	0.00	11.81	0.00	1.13	0.44 (1.99)	0.19
**Cas**	19 T, 13 S, 6 IM	0.00	37.87	0.00	26.10	3.46 (8.63)	0.82
**Cha**	5 T, 3 S, 2 IM	0.00	27.89	0.00	0.00	0.76 (3.94)	0.37
**Cis**	92 T, 56 S, 36 IM	0.00	40.50	0.00	34.42	15.96 (11.12)	1.05
**Cys**	46 T, 10 S, 36 IM	0.00	26.73	0.00	17.99	4.80 (6.71)	0.63
**Ech**	67 T, 44 S, 23 IM	0.00	41.00	0.00	37.93	13.17 (13.74)	1.30
**Eri**	35 T, 1 P, 15 S, 18 IM, 1 M	0.00	52.56	0.00	24.93	5.04 (9.54)	0.90
**Euc**	3 T, 1 S, 2 IM	0.00	17.78	0.00	0.00	0.23 (1.76)	0.17
**Gen**	6 T, 2 S, 4 IM	0.00	19.30	0.00	3.12	0.70 (3.15)	0.30
**Lav**	112 T, 23 P, 89 S	82.93	16.18	19.04	56.93	34.19 (12.52)	1.18
**Leo**	18 T, 2 S, 16 IM	0.00	21.15	0.00	8.64	1.44 (3.92)	0.37
**Lev**	1 T, 1 IM	0.00	11.75	0.00	0.00	0.10 (1.11)	0.10
**Med**	6 T, 1 S, 5 IM	0.00	16.40	0.00	2.17	0.52 (2.38)	0.23
**Pin**	2 T, 1 S, 1 IM	0.00	24.61	0.00	0.00	0.26 (2.37)	0.22
**Pru**	37 T, 11 S, 24 IM, 2 M	0.00	37.83	0.00	20.29	4.42 (7.99)	0.75
**Que**	7 T, 2 S, 4 IM	0.00	31.87	0.00	3.93	0.77 (3.76)	0.36
**Rub**	43 T, 11 S, 32 IM	0.00	30.06	0.00	17.99	4.70 (7.04)	0.67
**Tar**	19 T, 2 S, 16 IM, 1 M	0.00	17.96	0.00	11.83	1.55 (3.88)	0.37
**Thi**	5 T, 1 S, 4 IM	0.00	16.75	0.00	0.00	0.40 (2.19)	0.21
**Tri**	26 T, 4 S, 21 IM, 1 M	0.00	23.03	0.00	13.09	2.36 (5.04)	0.48
**Vic**	4 T, 4 IM	0.00	11.14	0.00	0.00	0.31 (1.67)	0.16
**Oth**	48 T, 29 IM, 19 M	0.00	10.30	0.00	6.71	1.65 (2.42)	0.23

^a^ The values of each parameter were obtained from the analysis of 112 honey samples.

^b^ T, Total samples; P, Predominant pollen (>45%); S, Secondary pollen (16 to 45%); IM, Important minor pollen (3 to 15%); M, Minor pollen (<3%).

^c^ Mean and Standard deviation (SD).

^d^ Standard errors of the means (SEM).

**Fig 5:**
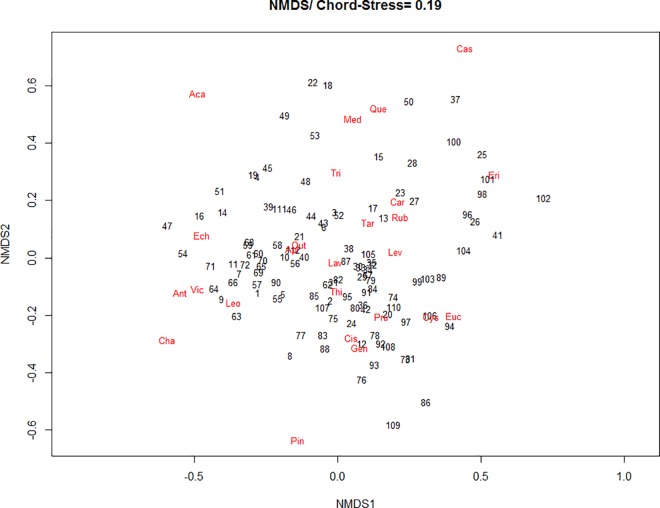
NMDS biplot of a chord distance matrix of the melissopalynological data in honey (stress = 0.19). Genera plants added using weighted averages. Sites: Mogadouro (1–10); Mirandela (11–20); Chaves (21–30); Castelo-Branco (31–40); Fafe (41–50); Lousã (51–60); Santarém (61–70); Odemira (71–80); Monchique (81–90); Montemor-o-Novo (91–100) and Amieira (101–112).

Although the pollen of *Lavandula* spp. was found in all samples, it had a higher percentage in those at the bottom of the ordination figure. The pollens of *Castanea sativa*, *Erica* spp. and *Rubus* spp. (secondary pollens in 13, 15 and 11 samples, respectively), and of *Quercus* spp. and *Medicago* spp. (detected in six and seven samples, respectively) are more closely associated with each other and with the samples located at the top of the ordination figure. Some pollen types, such as *Acacia* spp., *Chamaespartium* spp. and *Pinus* spp. had low frequency in the samples and lower contribution to the ordination (Fig 5).

We extracted two groups using the criterion of the silhouette width, from the result of cluster analysis of honey samples in relation to pollen types (%) found (Fig 3B). The cophenetic correlation coefficient was 0.75, which is reasonable as hierarchy representativeness factor (Sneath and Sokal, 1973).

**Fig 6:**
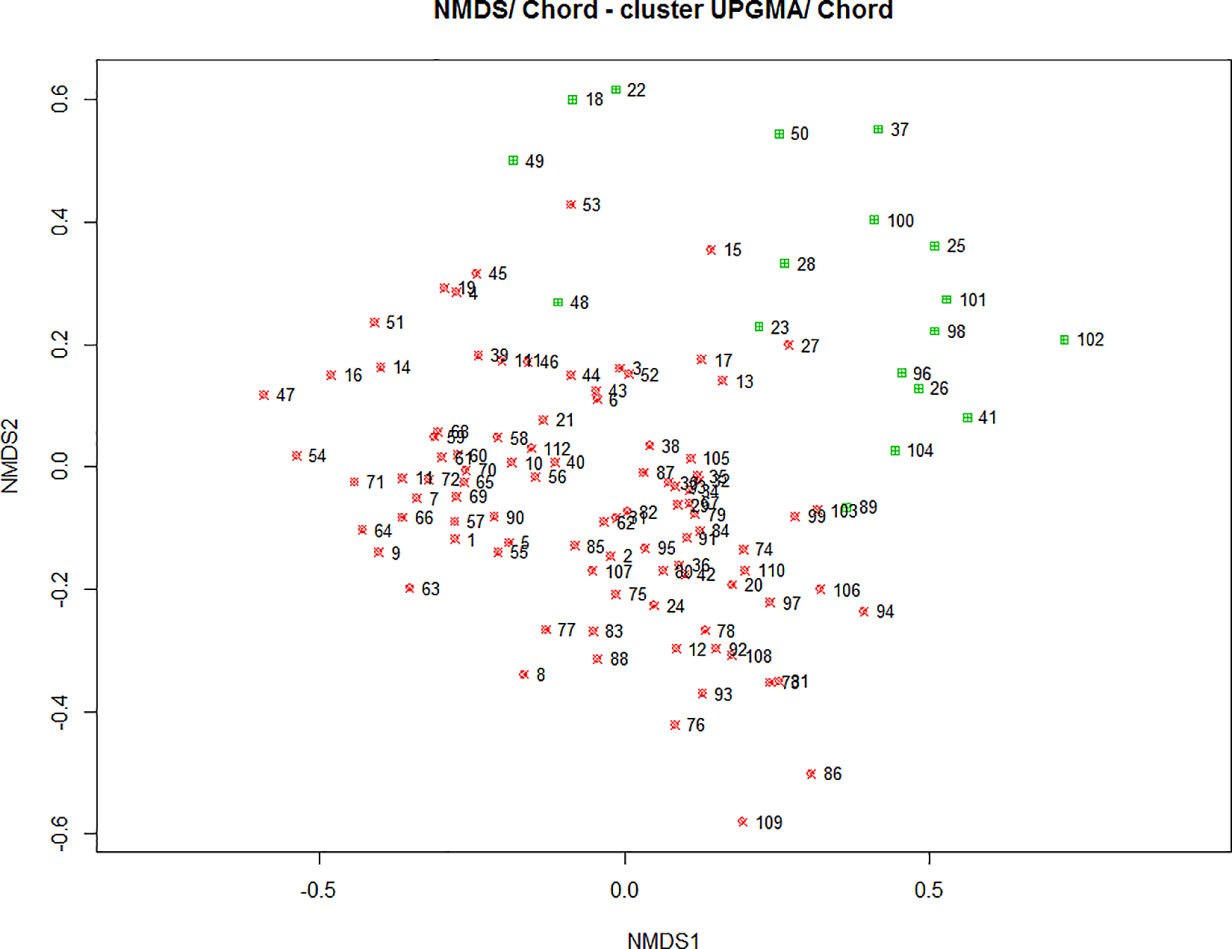
UPGMA clustering of a matrix of chord distance among sites in a NMDS ordination plot for melissopalynological data (CCC = 0.75). Sites: Mogadouro (1–10); Mirandela (11–20); Chaves (21–30); Castelo-Branco (31–40); Fafe (41–50); Lousã (51–60); Santarém (61–70); Odemira (71–80); Monchique (81–90); Montemor-o-Novo (91–100) and Amieira (101–112).

In Fig 6, it was verified a great similarity between the samples in respect to the percentage of pollen types found in honey, resulting in the formation of only two groups. A group of 18 samples, in green in Fig 6, represents the honey samples that had the lowest percentage of *Lavandula* spp. pollen, compared to the larger group in red. This same group had a greater association with *Erica* sp., *Rubus* sp. and *Castanea* sp., which were secondary pollens in many samples and pollen of *Medicago* sp. and *Quercus* sp., which were found in few samples and with a lower percentage in comparison to other pollen types (see Fig 5 and Table 5).

## Conclusion

It was observed great similarity between honey samples for most of the physicochemical parameters with the exception of flavonoids, phenols and proline. The percentage of *Lavandula* spp. pollen grains determined evidenced that all the honey samples under study were monofloral for this specie. The higher percentage of flavonoids, phenols and proline registered in some honey samples may be due to the presence of other secondary pollen species in the samples, particularly *Erica* spp., *Rubus* spp. and *Castanea* spp. Additionally, higher percentages of *Lavandula* spp. pollen grains were associated with superior sucrose contents. Furthermore, the sucrose content may be a parameter to assist in determining the botanical source of honey.

## Supporting Information

S1 Dataset Raw data for physicochemical analysis. (XLSX)

S2 Dataset Raw data for melissopalynological analysis. (XLSX)
